# Book Review:Prevention and Recovery from Eating Disorders in Type 1Diabetes:Injecting Hope

**DOI:** 10.1186/s40337-019-0233-7

**Published:** 2019-01-30

**Authors:** Phillipa Hay

**Affiliations:** Translational Health Research Institute, School of Medicine, Western Sydney University, Locked Bag 1797, Penrith, NSW 2751 Australia

“Good things come in tiny packages” exemplifies this 120-page volume written by Dr. Goebel-Fabbi [1]. It reflects her many years of clinical practice and wisdom as well as being embedded in empirical research and science. The first chapter is a narrative review of Eating Disorders as they occur in people with Type 1 diabetes mellitus (T1DM) with an introductory overview of both problems, predisposing risks and vulnerabilities, course and broad recommendations for treatment. A model of the interplay between cognitions, emotions, behaviors (notably insulin restriction) and physical/psychological symptoms (e.g. edema, depression) is presented.

The major content of the book is a series of 10 chapters presenting a thematic analysis of interviews of 25 women with T1DM who have experienced recovery (albeit not always complete remission) from an eating disorder. Each chapter takes a phase of people’s journeys from prevention, through seeking treatment and barriers, treatment experience, and life after treatment, including a chapter for people who continue to have eating disorder symptoms. The chapters follow a similar format, starting with emergent themes, and ending with a list of clinical learning points and a case study. There is an abundance of useful practice advice for both the eating disorder clinician and the diabetes specialist. A case in point is that commencing insulin is in itself an eating disorder “risk factor” as there is usually weight gain. A validating acceptance of dialectics common in people with eating disorders is outlined. Thus, rather than admonishing poor adherence, the clinician is encouraged to appreciate that the person can hold an extreme fear of weight gain (and insulin treatment) whilst sincerely wishing to have well controlled blood sugar levels and an improved metabolic status.

The book is very relevant for all clinicians working in either area, eating disorders or T1DM. Goebel-Fabbri highlights the paucity of research into the best management of comorbid eating disorders and T1DM. However, it is difficult to argue a randomized controlled trial is needed to support integrated care from eating disorder informed endocrinologists and diabetes informed eating disorder therapists. Whilst a single clinician may not often encounter the co-morbidity, when they do the challenges and potential for splitting of care are high. This book provides an important bridge across the knowledge divide.

People with lived experience would also find much in the book to help them navigate through treatment and recovery, particularly the accounts of people who have made good progress in their recovery journeys. As the title implies, these offer hope for others that they have a good chance as well to recover.

If I have a criticism it is that the book could be broader in context. For example it could have included consideration of these problems in men and people of other genders, and more resources outside North America. It is available through major online sellers and there is an electronic version on Kindle. It is not hard to obtain, well-written and should be widely read.
